# Editorial: Muscle Quality in Skeletal Muscle Function Deficit: Recent Advances and Potential Clinical and Therapeutic Implications

**DOI:** 10.3389/fphys.2022.847883

**Published:** 2022-04-04

**Authors:** Rosaly Correa-De-Araujo, Andrea P. Rossi, Mauro Zamboni, Odessa Addison, Iva Miljkovic, Bret Goodpaster

**Affiliations:** ^1^ National Institute on Aging, National Institutes of Health, U.S. Department of Health and Human Services, Bethesda, MD, United States; ^2^ Department of Medicine, Geriatrics Division, Ospedale Cà Foncello, ULSS2, Treviso, Italy; ^3^ Department of Medicine, Geriatrics Division, University of Verona, Verona, Italy; ^4^ Department of Physical Therapy and Rehabilitation Science, University of Maryland School of Medicine, Baltimore, MD, United States; ^5^ Department of Veterans Affairs and Veterans Affairs Medical Center Baltimore, Geriatric Research, Education and Clinical Center (GRECC), Baltimore, MD, United States; ^6^ Department of Epidemiology, University of Pittsburgh, Pittsburgh, PA, United States; ^7^ Advent Health, Translational Research Institute, Orlando, FL, United States

**Keywords:** muscle quality, myosteatosis, sarcopenia, mobility-disability, skeletal muscle function deficit

While current consensus-driven sarcopenia staging algorithms relate loss of muscle strength, muscle mass, and diminished physical functioning, there is a need to incorporate muscle quality and metabolic health into the larger concept of age-related Skeletal Muscle Function Deficit (SMFD). Factors underpinning muscle quality (e.g., fibrosis, fiber architecture/type, neural activation) play a critical role in decreased muscle function, impaired mobility, and metabolic dysfunction in older adults. Changes in muscle quality develop faster and appear earlier compared to changes in muscle mass. Recent attention has focused on changes in muscle composition based on myosteatosis (inter and intramuscular adipose tissue and intramyocellular lipids), which are known to adversely affect metabolism and peak muscle force generation and lead to a multitude of negative outcomes. The age-associated decline in lean body mass and the increase in myosteatosis are part of the development of several comorbid conditions in older adults that can result in physical impairment, frailty, and disability. (Correa-de-Araujo et al., 2017; Correa-de-Araujo et al., 2020).

Standardized approaches to assess the various aspects of muscle quality that influence major muscle functions and contribute to SMFD are needed. A rapid assessment of muscle composition across multiple clinical settings with minimal patient burden is highly desirable, but garnering consensus on the major domains of muscle quality and on measurement methods suitable for a variety of such settings are pending. This will help advance knowledge of SMFD and identify individuals who would benefit from interventions directed at improving muscle quality. (Correa-de-Araujo et al., 2017; Correa-de-Araujo et al., 2020).

This Research Topic holds manuscripts primarily using original studies including experimental research, with a focus on the biology, epidemiology, clinical or interventional research that document advances in knowledge of the role of muscle quality in aging related SMFD.

## Relevant New Findings in This Series

The mini review by Eshima discusses the impact of metabolic disorders, such as obesity and type 2 diabetes, on the regulation of (Ca2+)i in skeletal muscle. The author suggests that impairment of the (Ca2+)i flux in obesity and type 2 diabetes may contribute to skeletal muscle contractile dysfunction, which represents a reduction in muscle quality, and may be a promising target for a therapeutic approach for obesity- and type 2 diabetes-induced myopathy.

Poggiogalle et al.investigated the impact of protein intake in the context of an obesogenic diet on skeletal muscle function and intramuscular lipid infiltration. Adult and old male Wistar rats were randomly assigned to isocaloric standard or high protein diet and hypercaloric high fat with normal or high protein diet. The authors suggest that the effect of high-fat diet with enhanced protein intake (25%) may be linked to the reduced intramuscular fat seen in adult rats; this may have contributed to at least some preservation of skeletal muscle contractile properties. Although further investigation is needed on the high-protein diet’s potential role in preventing ectopic lipid deposition, high-protein intake seemed to mitigate the negative effects of a high-fat diet on skeletal muscle performance in adult rats but not in old rats. The authors relate these findings to lifespan changes in adult rats and their old counterparts in response to nutritional modulation of body composition and physical function.

Brown et al.evaluated muscle quality in the diaphragm muscle (DIAm) of rats. Muscle quality (specific force generation, oxidative capacity, mitochondrial abundance, and mitochondrial morphology) is distressed with age, exclusively in type IIx/IIb fibers. Aging does not seem to impact mitochondrial structure and function in type I and IIa DIAm fibers, as these continue to sustain breathing throughout life. In such fibers, mitochondria are abundant and more filamentous. In type IIx/IIb DIAm fibers, mitochondria are less abundant, more fragmented, and less functional. The authors suggest that mitochondrial fragmentation is likely a prime determinant of muscle fiber quality, and that interventions aimed to improving mitochondrial deficits in vulnerable fibers could prevent the age-associated decline in skeletal muscle quality.

Marron et al. document the need for separate examination of specific muscle groups when investigating fat infiltration. Data from the Multi-Ethnic Study of Atherosclerosis showed that associations differed between lipoproteins and intermuscular adipose tissue (IMAT) in the abdominal musculature, with the strongest relationships observed in the oblique muscles. Higher very low-density lipoprotein (VLDL) and lower high-density lipoprotein (HDL) were linked with higher muscle area, higher IMAT area, and lower IMAT density. The authors believe that such results are relevant to support additional studies seeking to establish whether the observed associations indicate a lipoprotein profile contributing to a decline in skeletal muscle quality and increased fatty infiltration with aging.

Rossi et al. opens a new area of research by investigating IMAT as a risk factor for mortality and muscle injury in critically ill COVID-19 patients. Cross sectional data suggest that higher levels of IMAT/muscle and low muscle density are associated with higher risk of intensive care unit (ICU) mortality and muscle injury. Muscle quality measurements from thoracic imaging performed during hospital stay could provide valuable information on health outcomes (length of stay, in-hospital mortality, or post-COVID-19 functional recovery). It was hypothesized that different factors, related to both intermuscular and intramyocellular adipose tissue deposition, can interfere with satellite cell activation and myoblast proliferation and differentiation, which are necessary steps to muscle repair after COVID-19 induced muscle injury. Intense inflammatory response in these patients leads to muscle strength deficit (upper and lower limbs), a phenomenon particularly relevant in obesity due to poorer clinical outcomes, higher mortality, and longer hospitalization. Additional longitudinal imaging and/or histological studies are critical to clarify the impact of COVID-19 induced inflammation on muscle damage in hospitalized subjects and the possible role of different muscle fat depots.

Clark et al. provide new insights into prior and current evidence on the potential use of electrical impedance myography (EIM) as a measure of muscle health in aging and geriatric medicine. EIM has been shown to reflect muscle health status (e.g., atrophy, fibrosis, and fatty infiltration) in a variety of conditions (e.g., developmental growth and maturation, conditioning/deconditioning, and obesity) and neuromuscular diseases (e.g., amyotrophic lateral sclerosis and muscular dystrophies). EIM is mainly limited by the impact of the subcutaneous fat layer, but it is a simple, broadly available and not invasive method that reflects information on muscle quality. The authors suggest that EIM measurements combined with machine learning approaches could support the development of prediction equations for common key outcome variables indicative of muscle health. They find EIM a promising technique to be used clinically alongside the most common assessment methods for muscle strength and mass as an indicator of muscle quality.

## Future Research

The field of muscle quality and its impact on aging related SMFD is still in its infancy. Although muscle quality has been included in the definition of sarcopenia (Cruz-Jentoft et al., 2019), no consensus on its evaluation and real meaning exists. Adipose tissue’s impact on skeletal muscle function and quality has gained substantial attention and shown to negatively affect several regulatory processes of muscle cells, with higher incidence of adverse outcomes in patients with sarcopenia and obesity. This special series highlights the importance of muscle characteristics in different settings and in relation to significant outcomes. The following are considerations for further research on muscle quality:

✓ Bridging molecular, pathologic, and population studies to identify what is being examined in the tissue and promote a better understanding of risk factors and mechanisms for myosteatosis, and whether the condition is reversible and to what extent.
✓ Generating new evidence on other biologic causative factors including reactive oxygen species (ROS) known to closely relate to aging muscle and to negatively impact muscle quality.
✓ Determining features of skeletal muscle characteristics that aid the sarcopenia and SMFD diagnosis including discriminating between patient groups and predicting incidence of metabolic disorders or disablement.
✓ Identifying best practices for the method(s) of acquisition for cross-sectional and longitudinal studies, data acquisition and analysis using the same cutpoints nomenclature, followed by crosstalk between imaging modalities to ensure what is being assessed.
✓ Identifying clinically feasible standardized imaging assessment tools to reliably evaluate muscle quality across a variety of settings. New validation studies remain essential to move toward a wider adoption of alternate imaging methods.
✓ Conducting longer longitudinal cohort studies to examine a variety of muscles across sex/gender and racial/ethnic groups, and disease populations. The identification of which muscles should be examined and measured (how), as well as the establishment of widely accepted cutpoints will likely enhance clinical practice (individuals at risk for poor outcomes).
✓ Determining the magnitude and significance of the associations between lifestyle risk factors such as objective measures of physical activity and sedentary behavior with muscle quality, particularly among older adults.
✓ As therapies for preserving muscle mass, such as myostatin inhibitors and irisin-activating compounds, are already in development studies focusing on the biological determinants of myosteatosis and other components of muscle quality may have potential therapeutic implications.
✓ Establishing multidisciplinary collaborations to examine combination treatments (diet, physical activity, and medications) essential to manage individuals at risk. Combined examination of interventions will likely result in more useful information and provide new treatment options.
